# Sperm motility and morphology changes in rats exposed to cadmium and diazinon

**DOI:** 10.1186/s12958-016-0177-6

**Published:** 2016-08-08

**Authors:** Maria Adamkovicova, Robert Toman, Monika Martiniakova, Radoslav Omelka, Ramona Babosova, Vladimira Krajcovicova, Birgit Grosskopf, Peter Massanyi

**Affiliations:** 1 Department of Botany and Genetics, Constantine the Philosopher University, 949 74 Nitra, Slovakia; 2 Department of Veterinary Disciplines, Slovak University of Agriculture, 949 76 Nitra, Slovakia; 3 Department of Zoology and Anthropology, Constantine the Philosopher University, 949 74 Nitra, Slovakia; 4 Institute of Zoology and Anthropology, Georg-August University, 37 073 Göttingen, Germany; 5 Department of Animal Physiology, Slovak University of Agriculture, 949 76 Nitra, Slovakia

**Keywords:** Sperm, Motility, Morphology, Rat, Cadmium, Diazinon

## Abstract

**Background:**

Humans are ubiquitously exposed to multiple environmental contaminants. Consequences of combined action on the reproductive system remain unknown. This study aimed to assess single and joint effects of cadmium and diazinon exposure on sperm quality parameters.

**Methods:**

Male adult Wistar rats were randomized into 4 groups of ten animals each. Group A was used as a control, animals from group B were exposed to cadmium (30 mg/L), rats from group C were administered with diazinon (40 mg/L), and rats from group D were exposed simultaneously to cadmium (30 mg/L) and diazinon (40 mg/L) via drinking water for 90 days. Sperm morphology and motility were evaluated using a bright field microscope and a computer-assisted semen analysis.

**Results:**

The percentage of motile spermatozoa and morphologically normal sperm was markedly reduced in rats from the group B. Rats from the C group showed an increase in velocity parameters, amplitude of lateral head displacement, decrease in beat-cross frequency, and an increase in abnormal sperm morphology. Simultaneous coexposure to cadmium and diazinon increased distance and velocity parameters, and amplitude of lateral head displacement. Reductions were observed in straightness, linearity, wobble, and beat-cross frequency. The decreased normal sperm morphology rates were related to defects of the sperm tail.

**Conclusions:**

Exposure to cadmium and diazinon at relatively low doses impairs sperm quality and can reduce male fertility. Cadmium and diazinon caused significant changes on sperm morphology with varying effects on motility patterns. These parameters were significantly higher in the group D as compared to the group C. The findings have important implications for reproductive risk assessment of combined exposures to multiple chemicals.

## Background

Cadmium (Cd) is a globally widespread toxic element which has no known biological function. Environmental pollution with Cd can produce a wide range of adverse health effects in both experimental animals and humans [1]. Apart from occupational exposure, food is the main source of Cd for the non-smoking population. The accumulation of Cd over time can severely damage the lungs, liver, kidney, bone, and reproductive system [2, 3]. Male reproductive toxicity induced by Cd involves various mechanisms such as direct effect on the testicular tissue, altered accessory sex gland secretions, resulting in decreased semen quality and indirect endocrine effects, causing suppression of steroid biosynthesis [4, 5]. Cd affects multiple cellular processes, including cell proliferation, differentiation, and apoptosis [6]. In the testis, disruption of intracellular junctions due to Cd toxicity on vascular system results in hemorrhage, edema, necrosis and germ cells damage [7, 8]. Another factor that may cause disruption of spermatogenesis in the testes is oxidative stress. The excessive production of reactive oxygen species (ROS) leads to decline observed in normal morphology, concentration, and motility of sperms [9, 10].

Diazinon (DZN) is an organophosphorus synthetic insecticide entering the environment from agricultural and household application of the chemical to control pest insects [11]. For the general population, dietary exposure to DZN residues, particularly in vegetables and fruits, is the most likely route of exposure. Following absorption into the body, DZN is bioactivated through desulfuration to oxygen analogues diazoxon, which is more toxic than the parent compound [12, 13]. The primary effects of DZN are mediated through an inhibition of acetylcholinesterase in the peripheral and central nervous systems [14]. DZN is one of the most important insecticides that are associated with decreased semen quality in men [15]. Organophosphate insectides alter male reproductive function inducing spermatogenic disturbances through hormonal or genotoxic mechanisms [16]. Furthermore, the induction of oxidative stress associated with defective sperm function and reduced male fertility has been observed after DZN exposure [17, 18]. Prolonged exposure to DZN alters semen quality and sperm chromatin, reduces sperm motility and viability, and increase sperm morphological abnormalities [19, 20].

Humans can be exposed simultaneously or sequentially to complex mixtures of environmental chemicals from numerous sources. Attention has recently been focused on adverse reproductive outcomes associated with widespread and permanent contamination by heavy metals and pesticides [21, 22]. However, the effects of potential interactions between different chemicals on the reproductive toxicity remain unknown [23]. Sperm motility and morphology parameters are very important semen characteristics [24]. Therefore, the present study was designed to determine the single and combined effects of Cd and DZN on sperm parameters in rats in relation to evaluate their potential interaction.

## Methods

### Chemicals

Cd in the form of cadmium chloride (CdCl_2_), with purity 96 %, was purchased from Reachem, Slovak Republic. Pestanal grade DZN, analytical standard (C_12_H_21_N_2_O_3_PS), with purity 99 %, was obtained from Sigma-Aldrich Laborchemikalien GmbH, Germany.

### Animals

The experiments were performed using male Wistar rats obtained from the accredited breeding and experimental laboratory (SK PC 50004, SUA Nitra). The rats were individually housed in plastic cages in an environment maintained at 20–24 °C, 55 ± 10 % humidity and 12/12 h cycle of light and darkness with access to food (feed mixture M3, Machal, Czech Republic) and drinking water *ad libitum*.

### Experimental design

In a 90-day study of oral toxicity, the 4 week old Wistar rats were randomly assigned into four groups of ten males each. Group A of untreated rats served as the control group. Rats in the group B were exposed to Cd at 30 mg/L in drinking water. Rats in the group C were administered with DZN at 40 mg/L in drinking water. Rats in the group D were treated with Cd and DZN together in combination of their identical doses in drinking water. The average daily dose of Cd in the B group was 1.01 mg per rat, the average estimated daily intake of DZN was 1.31 mg per rat in the C group. In the group D, the average daily oral intake of Cd was calculated as 0.96 mg and the daily DZN intake was 1.28 mg. Dose regimen, duration and route of administration in this study were based on previously described research to induce toxicity but not mortality in animals [25, 26]. The rats were observed daily for survival and clinical signs of toxicity. Individual body weights, food consumption, and water consumption were measured at weekly intervals. At the end of the treatment period, all animals were sacrificed and complete gross postmortem examinations were performed. Sperm were isolated from the left epididymis and sperm analysis was evaluated as previously described [24].

### Sperm motility analysis

To evaluate spermatozoa motility parameters a computer assisted semen analysis - SpermVision™ CASA System (MiniTüb, Tiefenbach, Germany) with Olympus BX 51 phase contrast microscope (Olympus, Tokyo, Japan) was used. Sperm samples were diluted with physiological solution (10 μl) and pipetted into a Makler Counting Chamber (depth 10 μm, Sefi Medical Instruments, Haifa, Israel) and immediately assessed. Within each of the measurement by the CASA system, motility parameters from minimum seven fields of Makler Counting Chamber were analysed and 1000 sperms were evaluated per sample. Motion parameters included the percentage of motile spermatozoa (MOT), the percentage of progressive motility (PROG), distance average path (DAP, μm), distance curved line (DCL, μm), distance straight line (DSL, μm), velocity average path (VAP, μm/s), velocity curved line (VCL, μm/s), velocity straight line (VSL, μm/s), straightness (STR, %), linearity (LIN, %), wobble (WOB, %), amplitude of lateral head displacement (ALH, μm), and beat cross frequency (BCF, Hz).

### Sperm morphology analysis

Assessment of sperm morphology was based on computerized techniques with PC morphometric software M.I.S. Quick Photo and using light microscope Olympus AX 70 Provis (Japan). After semen collection, samples were fixed with Hancock’s solution and smears were prepared. The slides were stained with Giemsa’s dye and submitted to analysis at x400 magnification. A total of 500 spermatozoa from each rat were examined and individually scored normal or abnormal, according to the strict sperm morphology criteria. The morphological abnormalities were divided into head and tail defects. Sperm abnormalities of the mid-piece were included as part of assessment of the sperm tail. The percentages of normal and abnormal shaped sperms were calculated.

### Statistical analysis

Differences were tested for statistical significance by one-way analysis of variance (ANOVA) and post hoc Scheffe’s test using SAS 9.2 Enterprise Guide 4.3 software (SAS Institute Inc., Cary, North Carolina, USA). Data was expressed as means ± standard deviation (SD) and were considered statistically significant when *P* < 0.05.

## Results

### Body weight, food and water intake

No clinical signs indicative of systemic toxicity were observed in any animal during the study. There were no significant differences in final body weight, weekly food and water intakes among control and experimental groups, as shown in Table 1.

**Table 1:** Body weight, food and water intake

Group	A	B	C	D
Parameters	Mean ± SD	Mean ± SD	Mean ± SD	Mean ± SD
Body Weight (g)	405.00 ± 52.65	426.67 ± 25.25	406.00 ± 24.70	427.78 ± 19.22
Food Intake (g)	155.17 ± 27.26	169.04 ± 30.56	147.32 ± 23.32	148.09 ± 21.35
Water Intake (ml)	254.49 ± 32.50	252.28 ± 31.41	239.63 ± 33.63	233.39 ± 31.61

*P < 0.05; **P < 0.01; ***P < 0.001

### Evaluation of sperm motility

Results of epididymal sperm analysis of the rats from all groups are summarized in Table 2. The sperm motility analysis showed significant changes in all motion parameters of rats from the group B. There was a significant decrease in MOT (*P* < 0.001) and PROG (*P* < 0.01). The results showed decreased distance parameters, including DAP (*P* < 0.01), DCL (*P* < 0.01), and DSL (*P* < 0.001). Similarly, parameters reflecting velocity characteristics demonstrated a significant reduction (*P* < 0.01). Cd also significantly decreased STR (*P* < 0.001), LIN (*P* < 0.001), WOB (*P* < 0.001), ALH (*P* < 0.05) and BCF (*P* < 0.001).

**Table 2:** Sperm motility analysis

Group	A	B	C	D
Parameters	Mean ± SD	Mean ± SD	Mean ± SD	Mean ± SD
MOT (%)	50.31 ± 9.15	16.72 ± 20.38***	39.87 ± 20.99	53.03 ± 8.58
PROG (%)	27.69 ± 7.34	6.97 ± 15.23**	20.00 ± 14.54	29.99 ± 6.85
DAP (μm)	21.95 ± 3.29	10.39 ± 9.61**	24.55 ± 3.57	32.32 ± 2.57***
DCL (μm)	33.91 ± 4.98	16.14 ± 14.57**	37.54 ± 5.41	55.31 ± 4.70***
DSL (μm)	17.65 ± 2.15	7.65 ± 6.36***	19.42 ± 3.37	24.34 ± 2.12***
VAP (μm/s)	52.73 ± 9.25	25.43 ± 24.69**	62.44 ± 9.34*	80.00 ± 6.37***
VCL (μm/s)	81.10 ± 13.88	39.57 ± 37.33**	95.01 ± 14.47*	134.74 ± 11.30***
VSL (μm/s)	42.37 ± 5.92	18.76 ± 16.28**	49.16 ± 8.52	60.50 ± 5.14***
STR (%)	0.81 ± 0.04	0.35 ± 0.24***	0.79 ± 0.04	0.75 ± 0.02**
LIN (%)	0.53 ± 0.04	0.24 ± 0.18***	0.53 ± 0.04	0.45 ± 0.02***
WOB (%)	0.65 ± 0.03	0.31 ± 0.23***	0.66 ± 0.03	0.60 ± 0.02***
ALH (μm)	4.52 ± 1.26	2.52 ± 1.93*	6.76 ± 1.06***	9.74 ± 0.69***
BCF (Hz)	22.37 ± 2.71	7.99 ± 5.79***	17.95 ± 2.73**	16.72 ± 1.85***

*MOT* motility, *PROG* progressive motility, *DAP* distance average path, *DCL* distance curved line, *DSL* distance straight line, *VAP* velocity average path, *VCL* velocity curved line, *VSL* velocity straight line, *STR* straightness, *LIN* linearity, *WOB* wobble, *ALH* amplitude of lateral head displacement, *BCF* beat cross frequency; *P < 0.05; **P < 0.01; ***P < 0.001

The sperm of rats from the group C showed significant increases (*P* < 0.05) in VAP and VCL, consistently with ALH (*P* < 0.001). BCF values were significantly decreased (*P* < 0.01). No significant differences were observed for MOT, PROG, VSL, LIN, STR and distance parameters.

Compared with the control group, there was no significant difference in MOT and PROG parameters in the group D. However, there was a marked increase (*P* < 0.001) in all distance and velocity parameters. Furthermore, the mean values for ALH increased significantly (*P* < 0.001). Conversely, significant decreases were detected in progression parameters VSL (*P* < 0.01), LIN (*P* < 0.001), and vigour parameters WOB (*P* < 0.001), and BCF (*P* < 0.001).

### Evaluation of sperm morphology

**Fig. 1:**
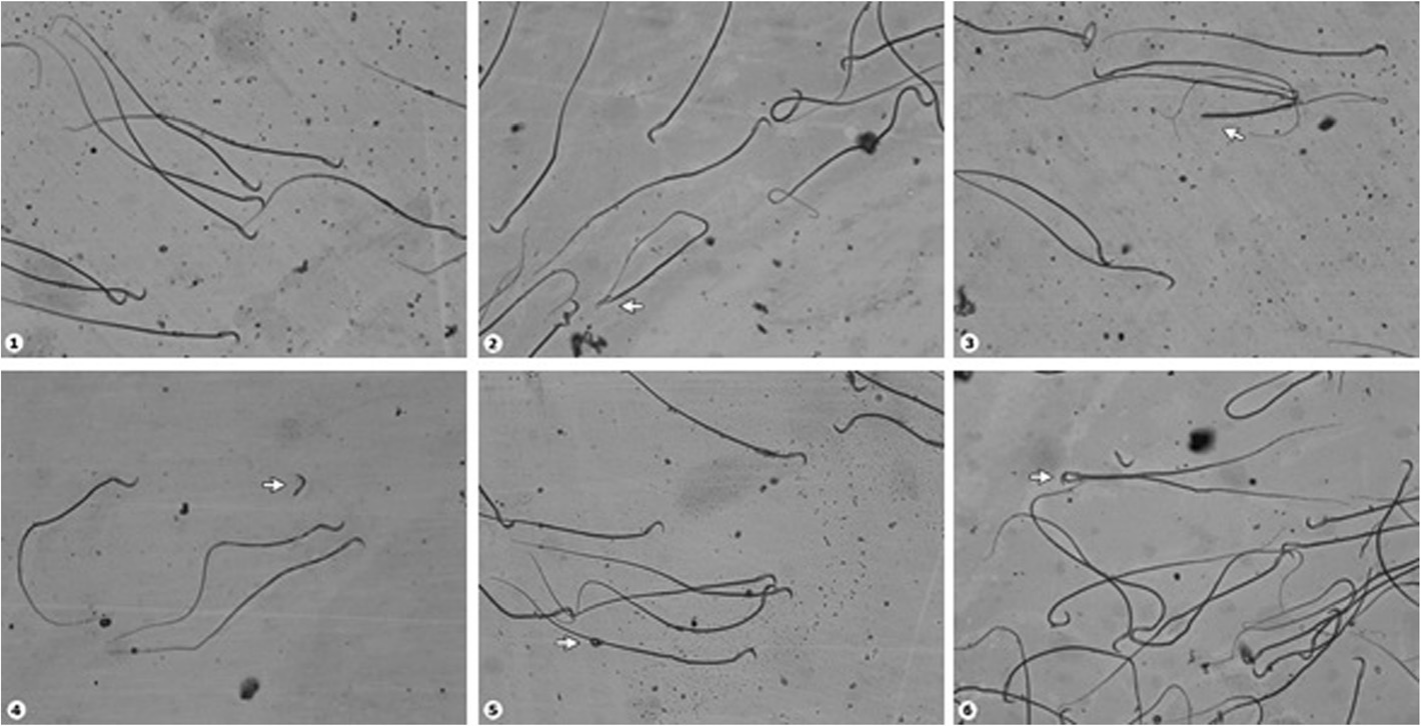
Microphotographs illustrating morphologically normal sperm and various sperm defects. 1 – Normal morphology; 2 –Bent neck; 3 – Headless tail; 4 – Detached head; 5 – Cytoplasmic droplet; 6 – Pairing phenomenon (Giemsa stain; original magnification × 400)

The percentages of normal and abnormal spermatozoa are shown in Table 3. Morphological analysis of semen samples revealed a significant lower percentage of spermatozoa with normal morphology in all experimental groups (*P* < 0.001). The significantly increased (*P* < 0.01) incidence of sperm with abnormal head in group B were detected. Moreover, the number of sperm head defects were significantly higher in the group C (*P* < 0.05). The common head defects include detached head and sperm with abnormal head number. Simultaneous coexposure to Cd and DZN did not produce any variation in the frequency of sperm head abnormalities. The percentage of sperm with normal tails significantly decreased (*P* < 0.001) in all exposed groups. The predominant types of abnormalities were sperm with knob-twisted flagellum and retained cytoplasmic droplets. The representative microphotographs of sperm morphology are shown in Fig. 1.

**Table 3:** Sperm morphology analysis

Group	A	B	C	D
Parameters	Mean ± SD	Mean ± SD	Mean ± SD	Mean ± SD
Normal Morphology	97.44 ± 0.94	91.09 ± 2.91***	91.60 ± 3.37***	94.56 ± 1.12***
Abnormal Head	1.74 ± 0.69	3.87 ± 1.64**	3.73 ± 2.49*	1.84 ± 0.61
Abnormal Tail	0.82 ± 0.39	5.04 ± 2.65***	4.67 ± 2.06***	3.6 ± 1.04***

*P < 0.05; **P < 0.01; ***P < 0.001

## Discussion

In the present study, results indicate significant decline in sperm motility and morphology of the rats exposed to Cd. CASA based sperm motion analysis depict a significant decline in all evaluated motility parameters. Furthermore, normal sperm morphology was significantly affected by increased percentage of sperm with detached head and increased abnormal sperm tail morphology. These findings are similar to those reported from both animal studies [27, 28] and research involving humans [4, 29]. The decrease in sperm concentration and motility, and the increase in dead and abnormal sperm of rats orally given CdCl_2_ at 5 mg/kg bw for 30 days were documented [27]. Similarly, subchronic exposure to CdCl_2_ at 40 mg/l for 30 days resulted in decreased sperm motility and impairment of spermatogenesis in rats [28]. Various mechanisms may explain reduced sperm quality induced by Cd. The alteration in sperm parameters could be attributed to direct effect on testicular tissue which leads to reproductive dysfunction such as reduced sperm count, motility and morphology [30]. Cd specifically disrupts Sertoli-germ cell tight junctions and thus leads to the failure of spermatogenesis. Profound testicular damage displays destruction of the seminiferous tubules and progressive sloughing of immature germ cells which result in abnormalities in early sperm development [8, 31]. Furthermore, low dose exposure to Cd affects steroid hormone actions involved in the regulation of reproductive processes. The maintenance of normal steroidogenic enzymes activity is required for proper testicular steroidogenesis and spermatogenesis. The decrease in sperm count and quality is correlated with decrease in testosterone levels and oxidative damage as evident from suppressed antioxidant enzyme activities [5, 32].

According to results from the CASA analysis, different motility patterns were identified after DZN exposure. Rats from the group C showed a significant reduction in BCF, but no apparent effect on MOT and PROG were found. Stimulation of motility was characterized by an increase of ALH and velocity parameters VAP and VCL. The percentage of morphologically normal sperm was lowered predominantly due to significantly higher number of sperm with abnormal tail morphology. Furthermore, significant increased incidence of abnormal detached sperm head was detected. These findings indicate that the exposure to DZN caused a severe disturbance of spermatogenesis, with a marked decline in sperm quality. Mice exposed to DZN at a dose 3 mg/kg/day for 6 weeks showed increased degenerate germ cells in seminiferous tubule and sperm morphological abnormalities [33]. Similarly, continuous oral administration of DZN to male rats at dose levels of 1.5 or 3 mg/kg/day for 65 days caused impaired reproductive function indicated by alterations in sperm motility, viability and morphology [34]. The induction of abnormal sperms was assumed to be a result of a direct effect of DZN on testicular tissue. The mice exposed to 4.1 and 8.2 mg/kg bw/day provoked severe alterations in the seminiferous tubules, including derangement and sloughing of the germ cells, the vacuolization of germ cell cytoplasm and the disruption of spermatogenic cells [16]. Although sperm DNA is normally resistant to aggressors because of its highly compacted structure, it has been proposed as a target of organophosphates [35]. Nuclear protamine phosphorylation caused by DZN exposure contributes to alterations in both sperm chromatin condensation and DNA integrity which has a negative impact on male fertility potential [19, 36].

Simultaneous coexposure to Cd and DZN displayed a higher incidence of hyperactivated-like motility than caused by DZN alone. The observed changes included significant increase in all measured distance, velocity parameters, and ALH. On the contrary, reductions were observed in progression parameters STR, LIN, and WOB, and vigour parameter BCF, while MOT and PROG were unchanged. Increased sperm velocity, increased ALH, and decreased LIN are known to reflect the increased vigor and asymmetrical movement of sperm tail which have been consequently related to hyperactivated motility [37]. Decrease in normal sperm morphology was linked to significantly increased sperm with abnormal tails. Interestingly, simultaneous coexposure did not produce any adverse effect on sperm head morphology. It is important to note that head and midpiece morphometric parameters may impact on motility patterns including VAP, VSL, LIN, STR, and BCF [38]. The possibility that DZN may cross the epididymal epithelium based on its lipophilic properties and reach the stored spermatozoa would explain its damaging effects on sperm structure and function [39]. It is known that combined exposure to pesticides and the heavy metals may alter toxic effects of the single compounds. A significant interaction between Cd and propoxur, carbamate insecticide, was detected on hematological, immune function and nerve conduction velocity [40]. Semen samples exposed to Cd, lead, chlorpyrifos, endosulfan showed reduced PROG, and acrosomal integrity. The fertilization capabilities of sperm were significantly reduced because of spermatozoa movement dysfunction [41].

In general, oxidative damage has been involved in the genotoxic and reproductive effects of various metals and organophosphorus pesticides. Cd and DZN toxicity involves formation of ROS, suggesting that oxidative stress plays a major role after their coexposure. It has been documented that combined exposure to arsenic and organophosphates result in increased levels of ROS in blood and tissues [42]. Increased lipid peroxidation has been correlated with alteration of sperm membrane, decreased sperm motility, and fertilization potential [27, 43]. Furthermore, DZN can alter semen quality and sperm DNA integrity or its associated proteins in the testis by generation of ROS which could intensify testicular dysfunction [18]. Increased rate of sperms with retained cytoplasmic residues may lead to increased ROS production and altered sperm quality [44]. Sperm membrane contains a characteristic high level of unsaturated fatty acids which makes spermatozoa particularly susceptible to oxidative damage [45]. Low levels of ROS produced by spermatozoa are needed for physiological processes involving sperm capacitation and the acrosome reaction. Excessive generation of ROS leads to reduced mitochondrial membrane potential and is associated with a decreasing energy availability, which may impede sperm motility [46, 47]. The most interesting aspect of this study was the fact, that concurrent administration of Cd and DZN elevated velocity and distance parameters together with ALH, in contrast with the decreased motility parameters than did Cd alone. The present findings have important implications for reproductive risk assessment in determining the interactive effects of combined exposure on sperm quality in the adult population. However, a better understanding of processes and pathways involved in the toxic actions of Cd and DZN is necessary and would require further experiments.

## Conclusions

The present study demonstrates that low doses of Cd and DZN impair sperm quality, and can reduce male fertility potential. Sperm motility was markedly decreased in rats exposed to Cd. Sperm distance, velocity parameters, and ALH were significantly higher in the combined group than caused by DZN given alone. The decrease in normal sperm morphology due to increased rate of sperm with head or tail defects was found after single exposure to Cd and DZN. Simultaneous coexposure increased sperms with abnormal tail morphology. The results of this study could be beneficial for evaluating reproductive health risks of combined exposure to multiple chemicals.

## Abbreviations

ALH, amplitude of lateral head displacement; BCF, beat cross frequency; CASA, computer assisted semen analysis; Cd, cadmium; CdCl_2_, cadmium chloride; DAP, distance average path; DCL, distance curved line; DSL, distance straight line; DZN, diazinon; LIN, linearity; MOT, motility; PROG, progressive motility; ROS, reactive oxygen species; STR, straightness; VAP, velocity average path; VCL, velocity curved line; VSL, velocity straight line; WOB, wobble
